# Predicting Dropouts From an Electronic Health Platform for Lifestyle Interventions: Analysis of Methods and Predictors

**DOI:** 10.2196/13617

**Published:** 2019-09-04

**Authors:** Daniel Hansen Pedersen, Marjan Mansourvar, Camilla Sortsø, Thomas Schmidt

**Affiliations:** 1 Liva Healthcare A/S Copenhagen Denmark; 2 Centre of Health Informatics and Technology The Maersk Mc-Kinney Moller Institute University of Southern Denmark Odense Denmark

**Keywords:** eHealth, patient dropouts, adherence, law of attrition, digital health, chronic disease, data mining, logistic regression, decision trees

## Abstract

**Background:**

The increasing prevalence and economic impact of chronic diseases challenge health care systems globally. Digital solutions can potentially improve efficiency and quality of care, but these initiatives struggle with nonusage attrition. Machine learning methods have been proven to predict dropouts in other settings but lack implementation in health care.

**Objective:**

This study aimed to gain insight into the causes of attrition for patients in an electronic health (eHealth) intervention for chronic lifestyle diseases and evaluate if attrition can be predicted and consequently prevented. We aimed to build predictive models that can identify patients in a digital lifestyle intervention at high risk of dropout by analyzing several predictor variables applied in different models and to further assess the possibilities and impact of implementing such models into an eHealth platform.

**Methods:**

Data from 2684 patients using an eHealth platform were iteratively analyzed using logistic regression, decision trees, and random forest models. The dataset was split into a 79.99% (2147/2684) training and cross-validation set and a 20.0% (537/2684) holdout test set. Trends in activity patterns were analyzed to assess engagement over time. Development and implementation were performed iteratively with health coaches.

**Results:**

Patients in the test dataset were classified as dropouts with an 89% precision using a random forest model and 11 predictor variables. The most significant predictors were the provider of the intervention, 2 weeks inactivity, and the number of advices received from the health coach. Engagement in the platform dropped significantly leading up to the time of dropout.

**Conclusions:**

Dropouts from eHealth lifestyle interventions can be predicted using various data mining methods. This can support health coaches in preventing attrition by receiving proactive warnings. The best performing predictive model was found to be the random forest.

## Introduction

### Background

Chronic diseases such as diabetes, heart disease, chronic obstructive pulmonary disease, and cancer are collectively responsible for more than two-thirds of all deaths and 75% of the health care budget spending in Europe [1]. The increasing prevalence and enormous economic impact of chronic diseases are a critical threat to health care systems. This necessitates new treatments that can effectively handle more people at a lower resource-to-outcome ratio. The application of mobile computing and communication technology in health care (denoted as electronic health [eHealth]) has introduced new possibilities in terms of improving efficiency and quality of care [2]. Despite several studies showing promising results in terms of outcomes such as weight loss [3] and behavior change [4], the evidence for long-term effectiveness, and especially how to retain patients in digital interventions, remains limited [5,6,7].

In any eHealth program, adherence is a key challenge, as a substantial proportion of patients stop using the application and thus drop out of the intervention program before its completion, referred to as *nonusage attrition*, or simply a *dropout* [8]. Recently, a dropout rate of 72% was reported in an eHealth intervention for adults with type 2 diabetes [9], and more generally, dropout rates up to as high as 83% are reported [10,11]. Studies have sought to identify predictors of dropout, but a consistent set of predictors has not yet been identified [12]. Previous studies have found engagement and participation in an online forum [13], depressive mood [14], age, gender, vocational education and employment status [15], disease severity, treatment length, and chronicity [11] to be related to attrition. Prediction of dropouts has been evaluated in multiple studies, in which many often have been offset in an educational institution setting where high dropout rates are also a great concern. Survival analysis [16], logistic regression, random forest, and other machine learning algorithms [17] are commonly applied to address this problem, using demographics and other characteristics to predict dropout. The documented high attrition rates from eHealth interventions make it an attractive case to apply similar methods to predict patients at high risk of dropping out. Furthermore, the literature on data mining and predictive methods in relation to attrition in eHealth settings is very limited, suggesting a lack of implementation and integration of these methods in the eHealth domain.

### Objectives

The aim of our study was to assess the variables and methods for predicting dropouts of patients with chronic diseases in a digital lifestyle intervention and review their applicability for implementation in an eHealth platform. We utilized self-reported data including patient-reported outcome measures (PROMs) submitted by chronic lifestyle disease patients in an eHealth intervention provided by the Liva Healthcare (LIVA) platform (Textbox 1). To assess the research question of *how self-reported data can be applied to address the challenge of attrition in an eHealth setting*, we evaluated the factors associated with dropout and applied logistic regression, decision trees, and random forest. We proposed how these models can be implemented to visualize the results as predictive warnings to reduce dropouts. In this way, the data are used to improve the eHealth intervention by supporting health professionals and enabling them to re-engage patients at high risk of attrition. As defined previously, we applied a broad definition of eHealth given the scope of the intervention under study that is targeting a wide range of patients. However, the challenge of attrition is relevant for most eHealth interventions for lifestyle change.

**Short description of the LIVA platform and intervention.**

LIVA is a digital platform designed to facilitate lifestyle changes for patients with chronic diseases. The platform is used by Danish municipalities. Patients have an initial goal-setting meeting with their coach and are introduced to the LIVA app that allows setting and registering health goals (eg, steps, weight, exercise, or diet), monitoring progress, dialog with the health coach by receiving advice and sending messages, and participation in an online forum. Health coaches access the platform through an internet browser and are able to proactively advise patients on a weekly-to-monthly basis based on their patients’ input in the platform. Personal data and health information are collected from the patients during the 3 to 12-month intervention program to provide the treatment service and for research purposes.

## Methods

### Ethics and Approvals

Only pseudonymized data for which patients had granted their consent to make them available for research purposes were used in this study. Consent was obtained explicitly in the sign-up flow before the patient’s use of the service. Liva Healthcare processes the data as the data processor, using the means and purpose defined by the data controllers, that is, Danish municipalities.

### Data Collection and Selection

The study was retrospective, applying data collected by Liva Healthcare from June 7, 2016, to March 21, 2018. For active users, the anchor time point for features was the date of data collection and for dropouts, we went back 4 weeks before the date of dropout (see next section). Data were extracted from a Microsoft SQL database and further processed in Alteryx. The dataset contains several unvalidated PROMs and sociodemographic information entered by the health coaches. Consequently, values for weight loss and body mass index (BMI) were filtered to remove extreme outliers and unrealistic values (weight differences of >3.5 kg/week on average for weight registrations over 30 days or more and BMI >100 kg/m^2^). The dataset was cleansed to only include patients who were referred to the platform by their doctor or municipality and showed commitment to the intervention by being properly set up in an advisory, received 3 or more advices from their coach, and had been active in the platform for at least 14 days (N=2684). A baseline of 14 days was selected as patients receive weekly advice in the beginning and should therefore receive their third advice on the fourteenth day of the program. Patients with *less* than 3 advices and 14 days of participation in the program were removed from the dataset as they had either not yet shown commitment to the intervention, signed up by a mistake, or merely signed up within the last 14 days of data collection. Thus, it is not known if these patients dropped out or never meant to use the service.

### Definition of Dropout

Generally, a large variation in the reporting and measurement of adherence is seen in previous literature [18]. For the objective of this study, it was relevant to look at dropouts as patients who commit to the intervention and thenceforward discontinue using the platform, consequently dropping out. We proposed a definition of dropout that aligns with Eysenbach’s characterization from 2005 [8] and other operationalizations [11]. Dropout is hence defined as *4 consecutive weeks of not performing any actions,* *for example,* *registrations or messages, in the platform*. The threshold for a dropout after 4 weeks of inactivity was defined based on the insight that less than 5.0% (117/2684) of the patients become re-engaged in the program after 4 weeks of inactivity. For 79.2% (2126/2684) of the patients, 2 weeks of inactivity equals dropout, and 84.61% (2271/2684) of them drop out after 3 weeks of inactivity. Furthermore, dropouts are limited to the active coaching period, which is a maximum of 12 months. This might be lower for some providers, for example, 3 or 6 months. Patients who are still active after 12 months will move to a *retention* phase, and they will thus not be considered as dropouts if they discontinue the intervention.

### Analysis of Dataset

To gain insight into the population and understand the factors associated with nonusage attrition, we performed several analyses of the users’ activity patterns by illustrating activity over time in the program for several subgroups of the population. We defined a formula for the current activity level in percentage based on these insights. Some descriptive user statistics of the population and analysis of predictors in *t* test and simple logistic regression models are provided to gain additional insights.

### Data Mining and Model Evaluation

The Cross-Industry Standard Process for Data Mining framework [19] was applied as an iterative data mining approach. This allowed for several iterations of the models to be developed as the knowledge of the population increased based on a better understanding for the dataset and end users (health coaches) who were able to provide feedback during each iteration.

Inspired by studies performed in an educational setting, logistic regression, decision trees, and random forest methods were applied to classify participants in the intervention into either dropouts or nondropouts based on specific characteristics.

We tested 11 variables that were well represented in the dataset as potential predictors of dropout: Gender, age group, provider of the intervention, period of intervention, BMI at the beginning of the intervention, weight loss, number of advices received, number of messages sent, total number of weeks with inactivity, and inactivity in the last 1 or 2 consecutive weeks. For weight loss, we required registrations over at least 30 days to be included. Less than 20% of the patients who registered had provided their educational status and zip code, because of the low quantity, these variables were not applied.

The variables in the final models for logistic regression were selected based on mixed backward and forward selection using the Akaike information criterion [20]. For decision trees and random forest, variables were selected inherently by the hyperparameters. The minimum number of records allowed for a split and a terminal node was set to 50 and 25, respectively. The maximum allowed depth in the final tree was set to 10 to avoid overfitting. The trees were pruned with a complexity parameter set to 0.01 to reduce the number of branches and the relative error.

To assess the quality of the 3 different models and to compare the predictive performance, the dataset was split into an 80% training and cross-validation set and a 20% test set. Owing to the relatively small size of the dataset, the training and cross-validation were performed using stratified 10-fold cross-validation. Stratification was applied on the target variable to ensure each fold was a good representative of the overall dataset distribution to reduce the bias and variance of the models. The best performing method was then applied to the 20% holdout test set that had never been seen by the model. The quality of the models was assessed based on the area under the curve (AUC) on the receiver operating characteristic (ROC) curve, the precision, and the accuracy. The goal was to have a high precision as the false negatives were the most critical to reduce in this scenario, that is, patients at high risk for dropout not identified as a potential dropout.

### Adoption and Implementation

The findings from this study have been diffused among the health coaches using the Liva Healthcare platform and prototype models have been implemented into the platform. Interviews were conducted with health coaches to adjust the models in terms of when and how warnings should be present. Feedback was continuously collected, and data were analyzed to assess dropout rate following implementation.

## Results

### User Statistics

The final dataset contained 2684 patients registered in the LIVA database. The population was characterized by a greater proportion of females (1943/2684, 72.39%) compared with males (741/2684, 27.6%). The majority of the population was in the age range of 40 to 59 years, and the average lifetime on the platform was 108 days. Overweight patients represent the largest treatment group, but patients might enter the program with one or more of 7 other diseases and possible comorbidities (other secondary disease). Additional characteristics of the population are provided in Table 1 (*advice received* and *messages sent* refer to the dialog between patient and health coach).

The intervention status for the patients was that 53.99% (1449/2684) had dropped out, 39.43% (1060/2684) were currently in active advisory, 3.7% (100/2684) had completed the intervention (finished intervention after >12 months), and 3% (75/2684) were in the retention phase (>12 months in program). More than 1 in 4 dropouts had occurred in the first month of the program (between day 14 and 31, n=388, 26.8% of dropouts; Table 2).

**Table 1:** A summary of the population from the final dataset included in the models.

Parameter (statistic)	Description
Sample size (N)	2684 patients
Number of providers (N)	18 different providers with between 13 and 581 patients ever in program
Gender (percentage distribution)	72.4% females and 27.6% males
Age (years), mean (SD)	48.6 (13.2)
Treatment groups (percentage distribution)	Overweight (85%), diabetes (17%), heart diseases (12%), chronic obstructive pulmonary disease (5%), stress (15%), cancer (1%), alcoholism (1%), smoking (6%), or another secondary disease (20%)
Days on platform (minimum, median, maximum)	14, 82, 595
Start body mass index (kg/m^2^), mean (SD)	33.6 (6.0)
Advice received (minimum, median, maximum)	3, 7, 99
Messages sent (minimum, median, maximum)	0, 3, 156

**Table 2:** Number of dropouts over the period of intervention.

Months of intervention	Number of dropouts
1	388
2-4	633
5-8	300
9-12	128

### Preliminary Analysis of Predictors

In total, a larger proportion of females (1069/1943, 55.02%) had dropped out compared with males (380/741, 51.3%), and for age groups, the highest dropout rate was found among the oldest (above 75 years; Table 3).

Characteristics for the patients in active advisory and the dropouts were assessed for the predictor variables of interest. Gender was found to be significant in a Welch 2-sample *t* test *(P=*.01). Dropouts had a slightly lower starting BMI than the active patients *(P=*.01). No major differences were seen in average age among the 2 groups *(P=*.60) nor average weight loss *(P=*.88). Large variations in the risk for dropout were found among the different providers of the intervention, varying from 7.3% to 87.0% in a simple logistic regression model.

**Table 3:** Percentage of dropouts distributed in age group and gender. The percentage indicates the proportion of dropouts for the patients in the specific age group and gender.

Age group (years)	Female, n (%)	Male, n (%)	Total, n (%)^a^
18-39	600 (53.72)	161 (51.60)	761 (53.18)
40-59	1040 (55.48)	395 (50.13)	1435 (54.01)
60-74	284 (52.11)	161 (50.31)	445 (51.39)
>75	19 (63.33)	24 (54.55)	43 (58.11)
Total	1943 (55.02)	741 (51.32)	2684 (53.99)

^a^Percentage of the total population of participants.

### Activity Analysis

**Figure 1:**
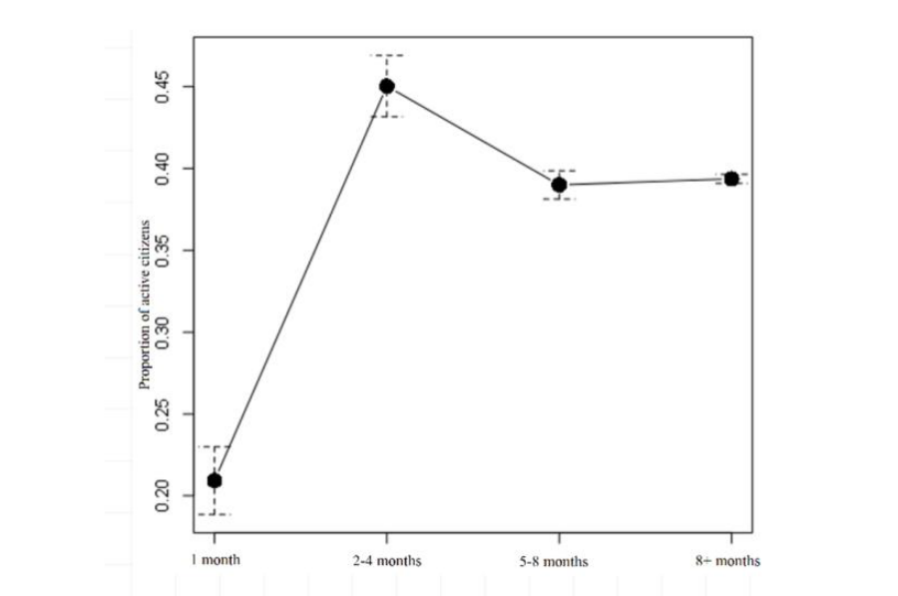
Proportion of active patients over 4 segments of the intervention period.

The largest proportion of active patients was in months 2 to 4 in the program and the lowest proportion was found at the beginning of the program in month 1 (Figure 1). The odds for a patient dropping out in the first month of the intervention were 4.35 times higher than for dropping out past month 8.

**Figure 2:**
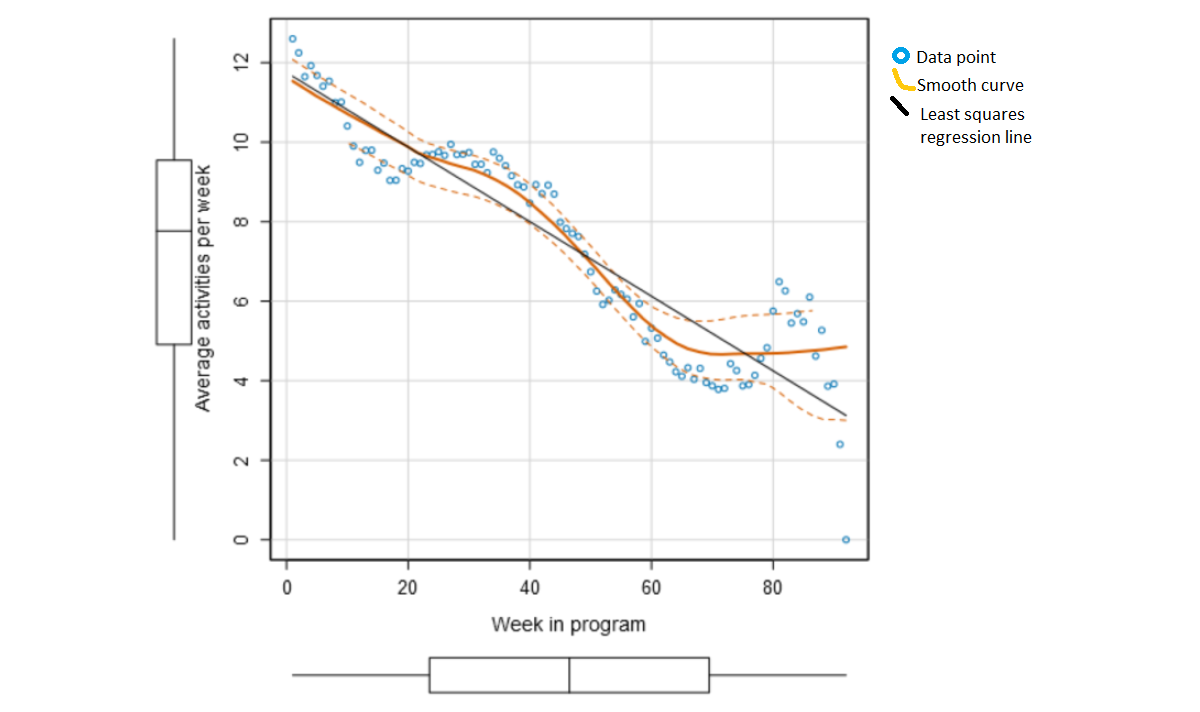
Average number of activities in the platform per week in the program for patients who either completed the intervention or entered retention (n=175), excluding week 0 in the program.

We analyzed if trends in the patients’ activity patterns could identify attrition by analyzing patient engagement in the platform over time. Evidently, patients who drop out have a very low level of activity (defined as a registration, forum posting, or messaging the coach) in the last weeks of their time on the platform. Overall, 71.77% (1040/1449) of the dropouts decreased their activity level by more than 50% in their last 2 weeks. However, there is also an expected decrease in activity that will occur over time, and individuals will have different trend lines for patterns in activity (Figure 2). The first week in the program (week 0) was found to have a significantly higher amount of registrations than the remaining weeks, on average 23.8 registrations, and was removed from the analysis to prevent skewing the linear regression line.

A variable for the patient’s current activity level defined in percentage of the baseline activity level was proposed (Equation 1). The variable accounts for (1) the patient’s average activity in the last 2 weeks, (2) the patient’s baseline activity (defined as the average activity in weeks 1-4 of the program), and (3) the regression line for the average activity levels over time (Figure 2). The average decrease in activities was found to be 0.094 per week. Patients with zero activity in the last 2 weeks will per default have a current activity level of 0%.

Current activity level (%) = Average activity last two weeks / (Baseline activity - (0.094 × weeks in program)) (1)

**Figure 3:**
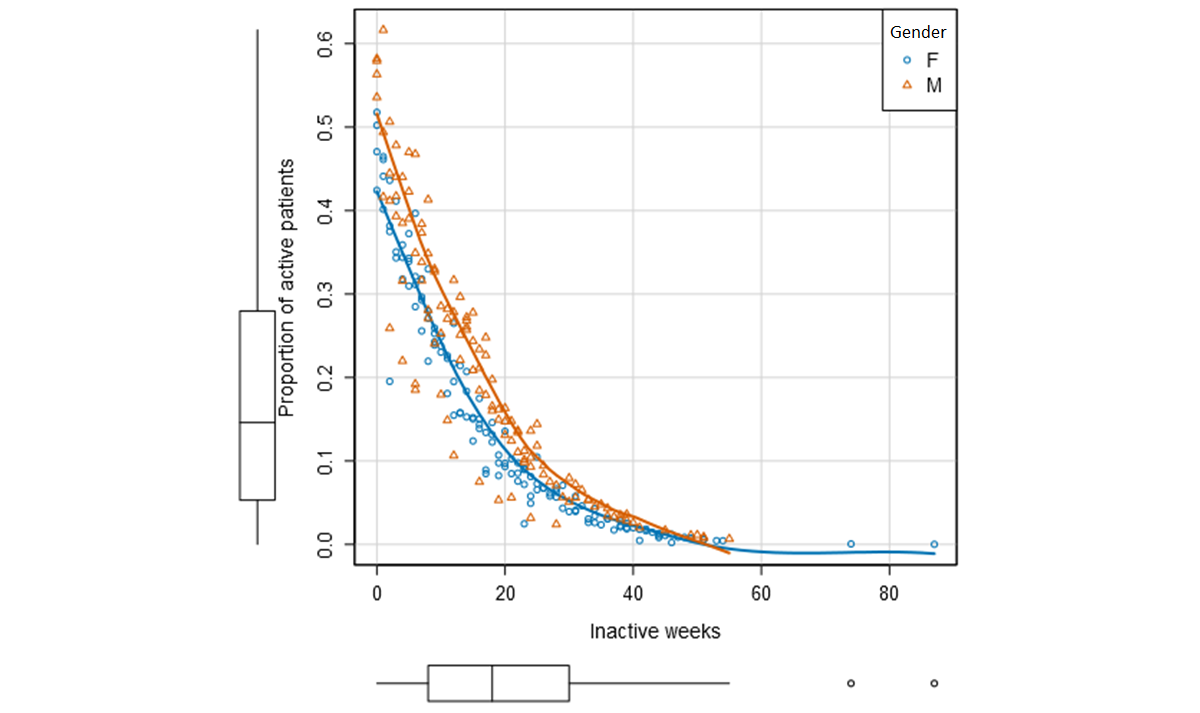
Proportion of active patients over the total number of inactive weeks in the program defined by gender.

**Figure 4:**
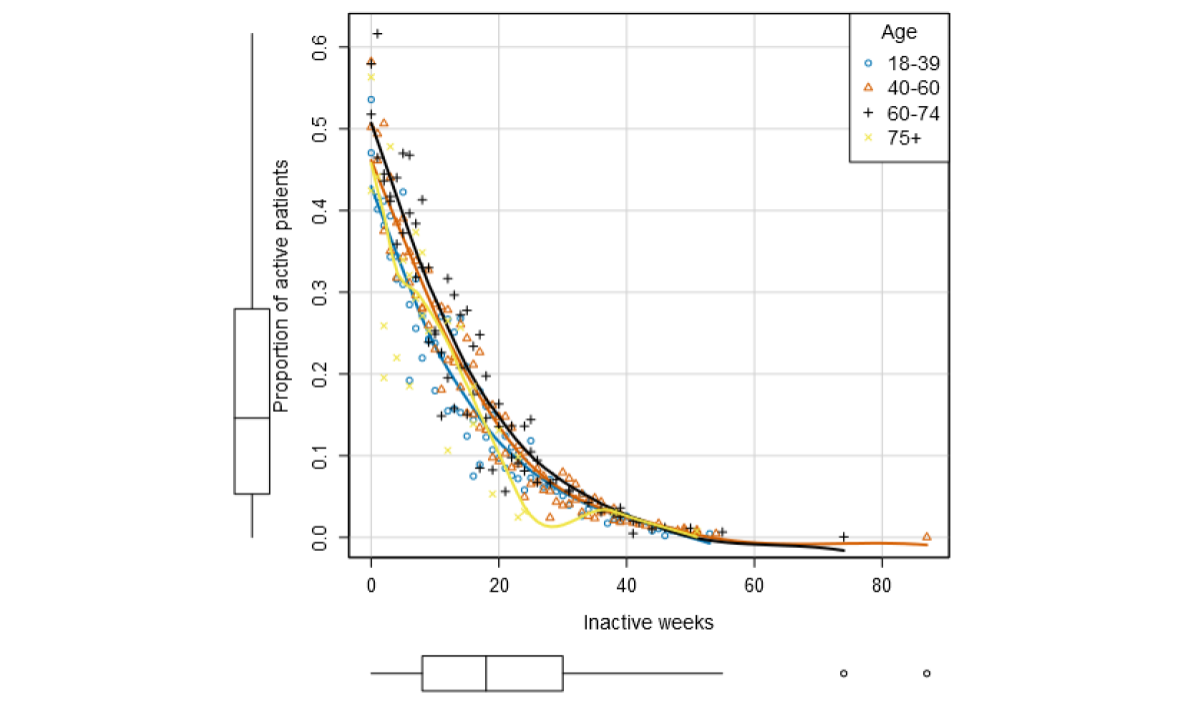
Proportion of active patients over the total number of inactive weeks in the program defined by age.

Fewer women tended to be active at the same number of inactive weeks compared with males, up to 40 weeks of inactivity, at which point of time, a very small percentage were still active in the program at the time of the data collection (Figure 3). For age groups, the oldest and the youngest age groups were the least active (Figure 4). The oldest group was also found to have the highest rate of dropouts (Table 3).

**Figure 5:**
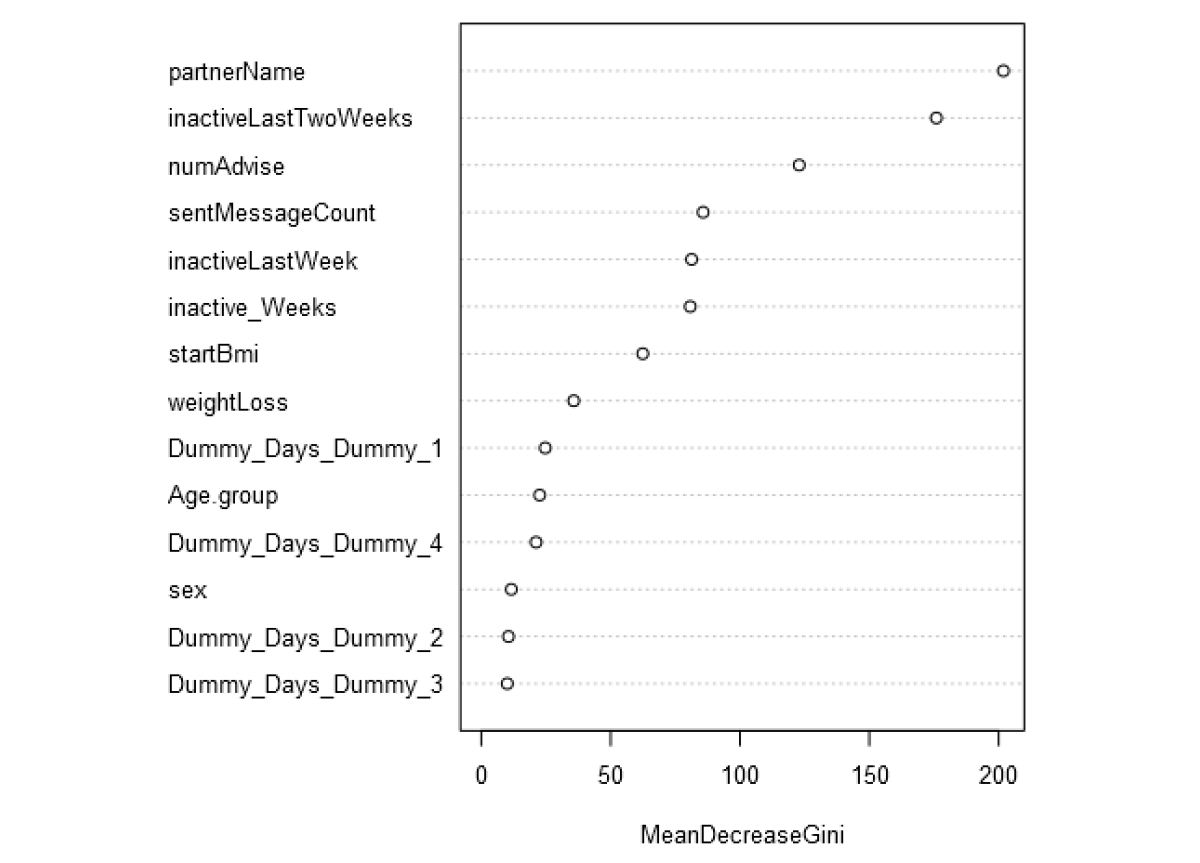
Variable importance plot for the 11 selected variables. Period of intervention is separated into 4 dummy variables.

Finally, introducing a variable that accounts for inactivity in the last 2 weeks of the program in any of the proposed models resulted in it becoming incomparably significant to any of the models’ other variables, except for provider of intervention (partnerName; see Figure 5). Hence, inactivity in the platform should be a critical warning for the health coach. A significant decrease in activity that deviates from the overall pattern might also be a critical sign for attrition and consequently an attention mark for the health coach.

### Model Selection for Dropout Prediction

**Figure 6:**
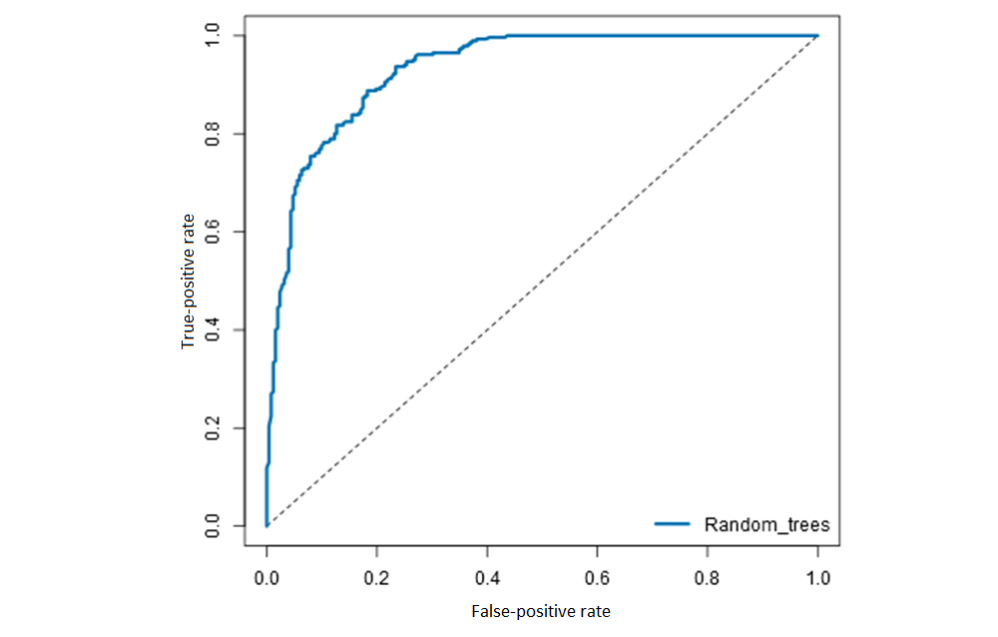
Receiver operating characteristic curve with area under the curve for the random forest model on the holdout test data.

The random forest achieved an AUC of 0.92 on the ROC chart and a Gini score of 0.84 on the stratified cross-validated training data, making it the best-performing model (Figure 6) compared with decision trees and logistic regression (Table 4). When applied to the holdout test data, the AUC increases by 0.01, and the model is thus not suspect to overfitting.

The precision of the random forest model was 0.89, with an overall accuracy of 0.86. This means that 89% of all dropouts were classified correctly as dropouts and 11% were mistakenly classified as active patients. This corresponds with 88.7% (253/285) dropouts in the holdout test data being classified correctly as dropouts.

**Figure 7:**
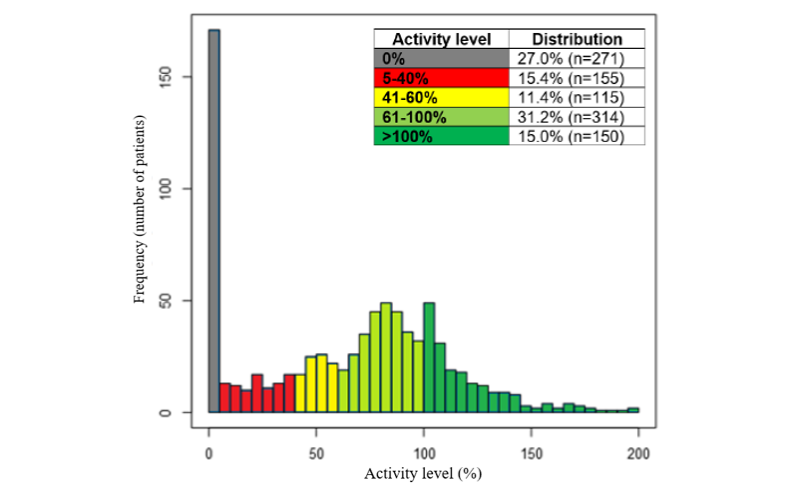
Histogram of current activity level (%; calculated using Equation 1) compared with forecasted activity based on the linear overall population trend line. Only patients with at least 6 weeks on the platform included

The outlined models for inactivity, activity level, and dropout risk have been implemented into the LIVA platform for the health coaches to be notified of attention markers related to patients that are at high risk of dropping out. The threshold values for activity levels have been set to highlight patients at medium (current activity level below 60% of expected) and high risk (below 40%), visualized with yellow and red warnings, respectively, for the health coach (Figure 7). The random forest model for dropout will show a yellow warning as the risk increases to above 60% and red if the risk is above 75%. The thresholds were selected based on an assessment of the patient distributions in collaboration with the health coaches.

**Table 4:** Area under the curve (AUC) and Gini index for the receiver operating characteristic on the 3 applied best performing models.

Model	AUC	Gini
Logistic regression	0.84	0.68
Decision trees	0.82	0.64
Random forest	0.92	0.84

## Discussion

### Principal Findings

This study applies real-world data from chronic lifestyle disease patients enrolled in an eHealth lifestyle intervention in municipal settings in Denmark. The findings show promising results in terms of applying data mining methods for the prediction of dropouts in eHealth interventions with high precision. To summarize, the following 4 key takeaway points were made clear in this study:

1. Patients are at the highest risk of dropout at the beginning of the intervention. Most dropouts occurred in the first part of the intervention, and evidence from other studies support the finding that when participants dropout, they do so early. For instance, 65% of dropouts from a diet and physical activity short message service text message program occurred within the first 2 weeks [21].
2. Attrition is not an abrupt process but something that happens over time. We found that patients reduce their activity in the platform significantly in the weeks leading up to their dropout. Therefore, being aware of abnormal decreases in activity should be a good indicator for health coaches to initiate re-engagement.
3. Dropout is primarily related to the program provider, outline of the intervention, activity in the platform (engagement), and, to a lower degree, the demographic variables available in this study. Multiple other studies have found attrition to be related to demographic variables [22,15], but these were not available in this dataset.
4. Predicting activity level and risk of dropout can enable personalized advices and goal settings. Our findings strongly suggest that dropouts can be predicted, and personalized coaching can be supported by several parameters. However, there are some limitations to the study that will be discussed in the following section.

### Limitations

The definition of dropout was based on patients not using the platform for 4 consecutive weeks. However, this might also include patients who stop using the platform because of having achieved their desired goal or goals, for example, behavior change or weight loss, or because the advisor has terminated the patient for other reasons. Termination reasons have been implemented in the platform following this study. In addition, the length of the intervention program offered by the provider was varying and unknown. This influences the likelihood of patients being incorrectly labelled as dropouts and should therefore be taken into consideration for the definition of the matter.

Dropouts in the first 14 days were excluded from this study because of large uncertainties in the reason for dropout. As this group of patients was very diverse and the amount of data were primarily limited to their sign-up registration, it provided a restricted dataset for analysis. This suggests a possibility for a future study to look into these very early dropouts.

The reliability of the findings is limited by some of the applied data (weight loss and BMI) being PROMs and most of the variables being self-reported by the patients. The data are, to some degree, validated by the health coaches, and extreme outliers are automatically marked as unrealistic by the Liva Healthcare system, but it has not been clinically verified.

The activity analysis was based on a simple linear regression despite the pattern being shaped as a higher-order polynomial. A simple approach was sufficient for the focus of this study; however, further investigations into activity patterns showed large variations in individual patterns. For future studies, time series analysis on the activity patterns of a larger population could potentially be the basis for interesting risk models that could analyze activity levels for distinct patient types.

### Adoption and Implementation

The insights obtained from our work are not sufficient to maintain engaged patients on the platform. The knowledge must be put into action to have an effect on the attrition rates. The discoveries must be diffused among the health coaches using the eHealth platform but should also be integrated into the system to support them to the highest degree possible.

The finding that dropout is not an abrupt process but something that happens over time underlines the importance of the health coaches being warned of dropout risk to actively attempt a prevention of attrition. From a practical computational perspective, a random forest is a computational and expensive model, and depending on the nature of the intervention, the frequency of registrations by the patients, and the technical setup, it might not be applicable. Nevertheless, if the model is not expected to change frequently, then calculations can be performed, for example, every night, and can be used as the daily baseline for dropout risk in the advisors’ overview. Otherwise, more simple models such as logistic regression might be preferred.

Previous studies have shown adherence to be closely related to the level of engagement in the platform, that is, by participating in an online forum [13]. Socioeconomic status, occupation, and educational status have shown to be related to dropout [15], but this type of data have not been available in sufficient quantities for this research. Diagnosis and condition should also be included in future models. Utilizing these data types would provide important information for the models and likely increase the accuracy and possibly make distinct patient profiles clear. Thus, this added data could be used to further enhance and individualize the models.

The provider of the intervention was found to be the most significant predictor of dropout, together with inactivity on the platform. This indicates that efforts toward preventing attrition should be targeted at providers to the same degree as patients. Provider-specific attrition models may perform even better than the generic approach proposed in this work. Further insights into the providers and their strategies are required.

Finally, to better validate the warnings that have been implemented into the platform, it requires a randomized setup or a less diverse population that is spread across multiple providers with varying programs. However, initial feedback from the health coaches is very positive, and the dropout rate for patients that have entered into the program after the date of data collection for this study is at only 19.3% (N=6402) compared with 54.0% for the population included in this study.

### Perspectives

This study contributes to the literature on adherence and nonusage attrition in eHealth by analyzing activity patterns, assessing various methods and predictor variables for predicting dropout in a chronic patient lifestyle intervention, and proposing some perspectives for implementation. We expect future research and development in eHealth to apply data mining methods in the process of tailoring information to patients in a higher degree to achieve personalized interventions as the field of digital health continues to evolve. Ongoing research is currently assessing how lifestyle interventions can be tailored to the individual patient [23], and as artificial intelligence is gaining ground within health care, we expect to see interventions, treatment, and guidance being selected based on the most suited for the specific individual patient profile in the future.

### Conclusions

It is possible to apply methods from data mining in the context of predicting dropouts in an eHealth setting. Stratified cross-validation shows that patients at high risk of dropout can be predicted with 89% precision using a random forest model. Computational simpler models, such as logistic regression, are applicable as well but might produce less precise predictions. The risk of dropout can be visualized as warnings for the health coaches, so they can attempt to re-engage the patient in their intervention before dropout. Initial assessment of the models implemented in an eHealth platform in use shows a decrease in dropout rate. Obtaining more rich data on educational status and socioeconomic factors in combination with a better delineation of dropouts would increase the quality of the models.

## Abbreviations

AUC: area under the curve
BMI: body mass index
eHealth: electronic health
PROMs: patient-reported outcome measures
ROC: receiver operating characteristic
